# Atypical Presentation of Hereditary Sensory and Autonomic Neuropathy Type IIB: A Family of HSAN IIB With Spasticity Presentation and Review on Sensory Neuropathies With Spasticity

**DOI:** 10.1002/ccr3.73764

**Published:** 2026-10-29

**Authors:** Farzad Ahmadabadi, Marzieh Babaee, Narjes Jafari, Mohadeseh Fathi, Diba Akbarzadeh, Amin Jazayeri

**Affiliations:** ^1^ Pediatric Neurology Research Center Shahid Beheshti University of Medical Sciences Tehran Iran; ^2^ Pediatric Neurology Department, Mofid Children's Hospital, School of Medicine Shahid Beheshti University of Medical Sciences Tehran Iran; ^3^ Physical Medicine and Rehabilitation Research Center Shahid Beheshti University of Medical Sciences Tehran Iran; ^4^ Student Research Committee, School of Medicine Shahid Beheshti University of Medical Sciences Tehran Iran; ^5^ Department of Medical Genetics Shahid Beheshti University of Medical Sciences Tehran Iran

**Keywords:** FAM134B, hereditary neuropathy, HSAN IIB, pyramidal signs, spasticity

## Abstract

A 10‐year‐old boy from a consanguineous family presented with progressive lower‐limb spasticity and a family history of painless, non‐healing trophic ulcers of the lower limbs. Genetic testing identified a homozygous FAM134B mutation, confirming HSAN IIB and expanding its clinical spectrum with early pyramidal involvement.

## Key Clinical Message

Consider HSAN IIB in patients with early progressive lower‐limb spasticity especially in consanguineous families or when a family history of painless ulcers exists because FAM134B mutations can present with predominant pyramidal signs before clear sensory deficits appear.

**Abbreviations**
ACMG: American College of Medical Genetics and Genomics
EMG: electromyography
ER: Endoplasmic Reticulum
HSAN: hereditary sensory and autonomic neuropathy
HSP: hereditary spastic paraplegia
MRI: magnetic resonance imaging
NCS: nerve conduction study

## 1 Introduction

Hereditary sensory and autonomic neuropathy (HSAN) comprises a group of rare disorders characterized by dysfunction of sensory and autonomic neurons. Traditionally classified into five subtypes (HSAN I‐V) based on age of onset, clinical presentation, and genetic inheritance. The discovery of additional causative genes has led to an expanded classification, now encompassing HSAN I‐IX [1]. Although the global prevalence of HSAN2 remains unclear, it has been reported to affect both sexes equally, with symptoms typically emerging from birth or early childhood [2].

HSAN Type II is an autosomal recessive disorder that has four subtypes (A, B, C, D) and is caused by mutations in the *WNK1, FAM134B, SCN9A*, and *KIF1A* genes, primarily affecting sensory function and autonomic regulation [3]. Patients with HSAN IIB (MIM#613115) exhibit pronounced autonomic dysfunction, with or without distal motor involvement, setting them apart from HSAN IIA. In contrast, individuals with HSAN IIC present with distal muscle weakness, while those with HSAN IID show insensitivity to pain despite otherwise normal sensory modalities. Notably, HSAN IIB typically manifests in infancy or early childhood, with symptoms such as distal numbness and a progressive loss of pain, temperature, and touch sensations [3]. However, spasticity is not a recognized feature of HSAN II, especially as the first presentation of the disease; therefore, making cases that present with both HSAN III and spasticity particularly unusual and warranting further investigation [4].

This family report aims to describe a rare presentation of HSAN IIB in a patient exhibiting spasticity, a feature not typically associated with the disease. By detailing the clinical findings, diagnostic workup, and possible underlying mechanisms, we highlight the need for further research into atypical manifestations of HSAN II and their implications for diagnosis and management.

## 2 Case Presentation

### 2.1 Case History and Examination

A 10‐year‐old boy, born to consanguineous parents (first cousins), was referred to our clinic due to progressive lower limb spasticity and gait abnormalities. His developmental milestones were normal until Age 6, when he began to exhibit motor regression, including toe‐walking and mild knee contractures. He had achieved independent ambulation at 15 months.

On clinical examination, spasticity and hyperreflexia were observed in the lower limbs. Sensory examination revealed impaired vibration sense extending to the elbows in the upper limbs and to the knees in the lower limbs while light touch, pain, and proprioception were preserved. Coordination testing showed normal finger‐to‐nose performance but slow heel‐to‐shin movement. Verbal and cognitive development was normal. The patient also reported urinary and fecal incontinence, which, according to the family, had been present since early childhood. Apart from urinary and fecal incontinence, no other systemic complaints were reported. The index patient had no history or examination findings of cutaneous ulceration, painless injury, self‐mutilation, or other trophic skin changes.

## 3 Methods

### 3.1 Differential Diagnosis and Investigations

Given the progressive spastic gait, the initial diagnostic workup included brain and spinal MRI. Brain imaging was unremarkable, but spinal MRI revealed a mild prominence of the central spinal canal, most notable in the distal thoracic segment. Based on the clinical presentation—spasticity, hyperreflexia, and gait disturbances, hereditary spastic paraplegia (HSP) was initially suspected. As part of the broader differential diagnosis, electrophysiological studies (EMG (electromyography)/NCS (nerve conduction study)) were performed due to impaired vibration sensitivity in the physical exam. The NCS showed lower limit normal sensory amplitude responses with normal motor nerve conduction recorded and EMG findings.

Because of family history of 3 s cousins with adolescent‐onset skin ulcers and a prior abortion of a fetus carrying the same gene mutation, genetic testing was pursued (Figure 1).

**FIGURE 1:**
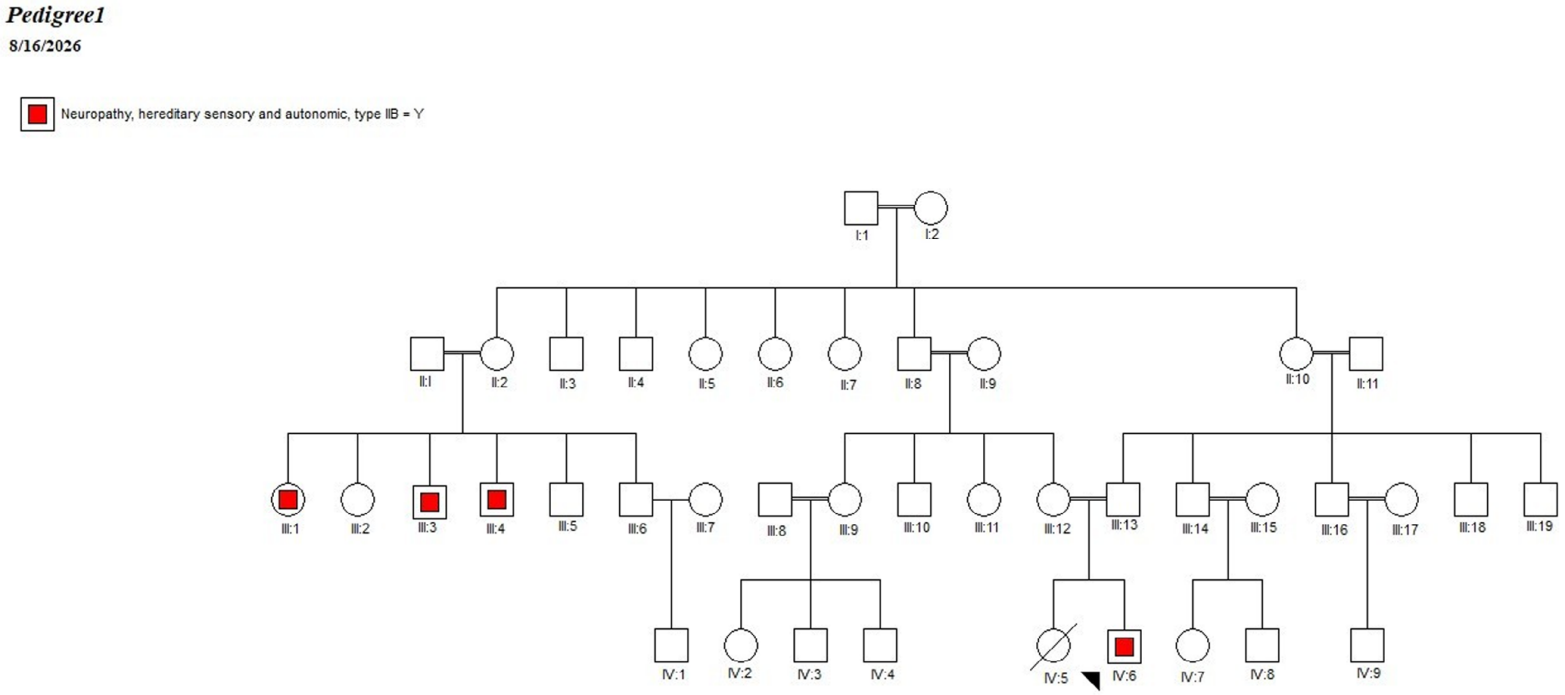
Pedigree of the family with hereditary sensory and autonomic neuropathy Type IIB (HSAN IIB). Squares represent males, circles represent females, and red‐filled symbols indicate affected individuals. The arrow identifies the index patient (IV: 6). The diagonal line indicates pregnancy loss or death, and the double horizontal line denotes consanguinity.

This revealed a homozygous pathogenic mutation in *FAM134B (RETREG1)*, confirming the diagnosis of hereditary sensory and autonomic neuropathy Type IIB (HSAN IIB). Both parents were found to be heterozygous carriers (Table 1, Figure 2). Following genotype–phenotype correlation, a follow‐up nerve conduction study was performed, which showed decreased sensory amplitudes—suggestive of an evolving sensory neuropathy consistent with HSAN IIB.

**TABLE 1:** Whole exome sequencing of patient and his parents.

ID	Gene and transcript	Variant	Zygosity	ACMG (American College of Medical Genetics and Genomics) classification	Inheritance
Patient	*FAM134B* NM_001034850 (*RETREG1*)	c.458+2T>C	Homozygous	Pathogenic	Autosomal recessive
Mother			Heterozygous		
Father			Heterozygous		

**FIGURE 2:**
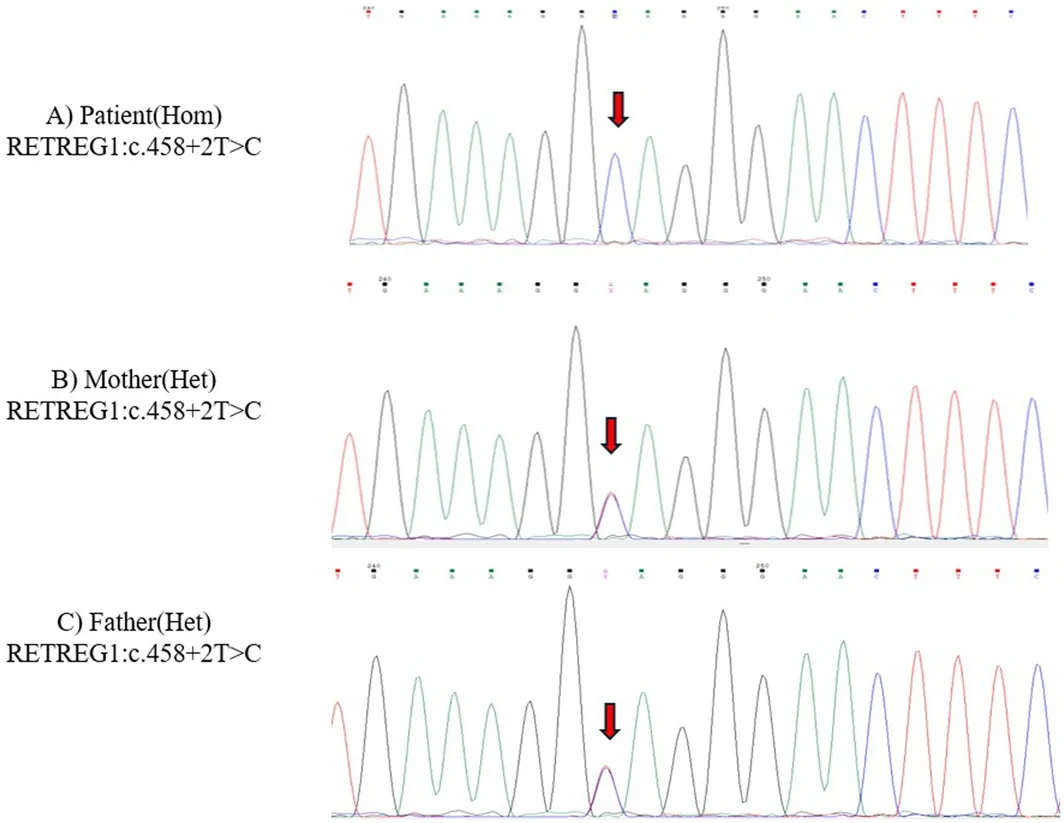
The results of Sanger sequencing. Confirmation of the variant was performed in the patient and his parents via sanger sequencing. (A) The patient is homozygous for the pathogenic variant (c.458+2T>C) in the RETREG1 gene. (B) The patient's mother is heterozygous for the pathogenic variant (c.458+2T>C) in the RETREG1 gene. (C) The patient's father is heterozygous for the pathogenic variant (c.458+2T>C) in the RETREG1 gene.

### 3.2 Treatment and Follow‐Up

Baclofen 10 mg daily was prescribed for spasticity; however, no significant improvement was observed after 4 months, and he is currently undergoing occupational therapy while increasing baclofen treatment (20 mg daily).

### 3.3 Examination of the Affected Relative

Additionally, one affected second cousin, who was 38 years old at the time of evaluation, underwent clinical assessment. According to the family, two other second cousins had a similar history of painless trophic ulcers beginning during adolescence; however, they were unavailable for direct examination, and detailed clinical and genetic information was not available. The symptoms in the affected second cousins started during adolescence at the age of 14 years. In this relative, the clinical course was characterized by progressive distal trophic changes of the lower limbs, culminating in deformity and self‐amputation (Figure 3). On examination, there were deformities and self‐amputation in the lower limbs, while the upper limbs were grossly normal with some ulcers. The touch and pain sensations were absent in the lower limbs up to the knee, while these sensations were normal in the upper limbs; proprioception in the upper limbs was normal, and vibration sense was decreased from distal to proximal segments. Neurological examination of this cousin demonstrated normal upper limb strength with a slight reduction (graded 4/5) in the lower limbs, deep tendon reflexes graded as 2+ in the upper limbs. Muscle tone in the lower limbs was increased, and spasticity was present, particularly in the distal segments. According to the family, he lost independent ambulation at the age of 16 years and has been wheelchair‐bound since then. At 24 years of age, he underwent amputation of right lower limb due to severe, non‐healing trophic ulcers and osteomyelitis, and approximately 10 years later, the contralateral limb was also amputated for similar complication.

**FIGURE 3:**
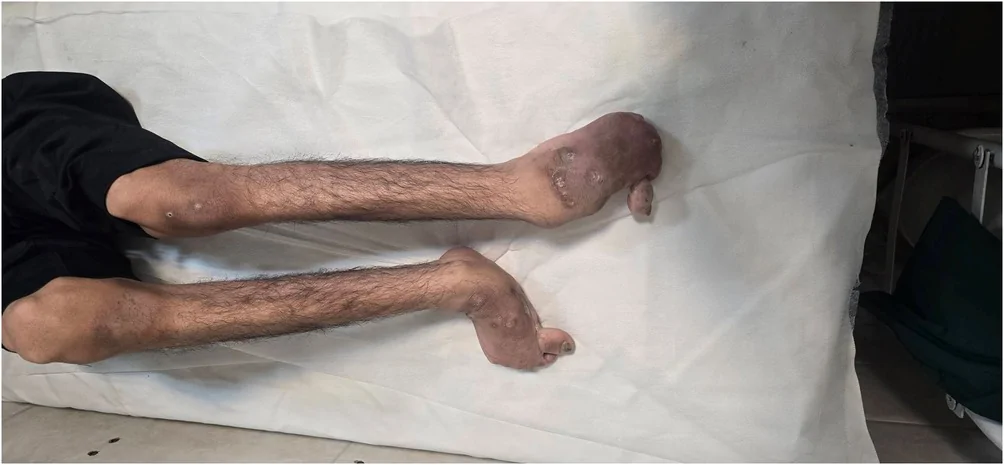
Clinical photograph of the affected second cousin with HSAN IIB. The image demonstrates marked deformity of the residual limbs and distal trophic skin changes, including ulceration and scarring resulting from recurrent painless injuries and non‐healing ulcers.

The presence of spasticity and central nervous system involvement in the index patient, alongside the variable sensory and motor findings in affected family members, challenges the conventional understanding of HSAN IIB as a purely sensory neuropathy and suggests a possible overlap with motor system involvement.

## 4 Discussion

HSAN IIB is a rare genetic disorder characterized by early childhood onset of distal sensory impairment, often leading to ulcerations, and is associated with variable autonomic features such as hyperhidrosis and urinary incontinence [3]. The condition primarily affects sensory neurons, leading to a loss of pain, temperature, and touch sensations, which can result in painless injuries and chronic skin ulcers [5].

Although sensory neuropathy is a predominant feature in HSAN IIB, spasticity is not typically associated with this condition. Therefore, the co‐occurrence of spastic paraplegia in individuals with HSAN2B is exceptionally rare, with only a few reported cases globally [6]. This overlap between sensory neuropathy and spastic paraplegia presents a diagnostic challenge, as it blurs the lines between HSAN IIB and complex forms of HSP, which can also exhibit sensory deficits alongside spasticity. However, unlike HSAN IIB, HSP primarily affects motor pathways and does not typically involve sensory loss or autonomic dysfunction. Accurate diagnosis is crucial for appropriate management and genetic counseling, highlighting the need for comprehensive clinical evaluations and genetic testing in such overlapping syndromes.

Besides, it is important to consider other potential mimickers such as cerebral palsy, spastic diplegia, or other hereditary neuropathies when evaluating such patients, particularly in early stages when clinical features may overlap [7].

*FAM134B* mutations, were first identified in 2009. *FAM134B* gene, also known as reticulophagy regulator 1 (*RETREG1*), is a protein coding gene which is located on 5p15.1 chromosomal region [8, 9]. The *RETREG1* gene encodes a protein that has a significant role in the cellular process known as autophagy. This process is a highly regulated mechanism that allows cells to maintain homeostasis by degrading and recycling cellular components [10, 11]. One of the key roles of the RETREG1 protein is to facilitate the autophagic degradation of the endoplasmic reticulum (ER), an essential organelle involved in the synthesis, folding, modification, and transport of proteins [11, 12]. Recent studies link *RETREG1* impairment to various diseases, including neuropathy, viral infections, vascular conditions, inflammation, and cancer [13, 14, 15]. Notably, *RETREG1*‐related neuropathies may be associated with viral infections due to its role in ER‐phagy regulation. Additionally, *RETREG1* is crucial for key biological processes like the cell cycle, proliferation, apoptosis, and metastasis, and is involved in the PI3K/AKT and WNT/β‐catenin signaling pathways [16, 17, 18].

Mutations in this gene are associated with hereditary sensory and autonomic neuropathy Type IIB (HSAN IIB) and are passed down in a family through an autosomal recessive pattern. Furthermore, several alternatively spliced transcript variants that generate multiple isoforms have been identified for this gene [19]. In our study, a patient with a homozygous pathogenic variant in the *RETREG1 (FAM134B)* gene was reported. This variant (c.458+2T>C) has been classified in the ClinVar database as pathogenic. Most of the reported HSAN2 IIB cases due to *FAM134B* mutations, involve Middle Eastern populations (Table 2). Several previously reported cases of HSAN IIB have demonstrated a range of clinical features with varying degrees of sensory, autonomic, and motor involvement. Our patient, as well as his extended relative (with limited data available), contributes to the understanding of this phenotypic variability.

**TABLE 2:** Clinical and paraclinical parameters among our patient and other studies.

Study, year (patient)	Kurth et al. [15] P1, 2, 3	Murphy et al. [20]	Aydinlar et al. [21]	Wakil et al. [6] F1, F2	Campos et al. [22] F1, F2	Tasdelen et al. [5]	Our patients F1, F2
Origin	P1: Saudi Arabia P2: Turkey P3: Italy P4: Dubai	Saudi Arabia	Turkey	F1, F2: Saudi Arabia	F1: Turkey F2: Portuguese	Turkey	F1, F2: Iran
Consanguinity	P1: + P2: same village P3: NR P4: +	+	+	F1, F2: +	F1: + F2: −	Same village	F1, F2: +
Age of onset	P1: up to 20 year old P2: up to 10 years old P3: first years P4: 10–20 years	Five	Eight	F1: one to 3 years F2: 4 years	F1: 9 to 12 years F2: 13 years	Six to 7 years	F1: 6 years, F2: 14 years
Ulcerations	P1: + P2: + P3: +	+	+	+	+	+	F1: − F2: +
Sensory function	P1: Impaired P2: Impaired temperature and sensation P3: Impaired	Sensory neuropathy	“Hypesthesia and hypoalgesia”	F1, F2: Sensory loss	F1: Thermo analgesia and proprioceptive hypoesthesia F2: Thermo‐algic and proprioceptive deficit	Loss of pain and light touch sensation	F1: Vibration sense impaired (upper limbs up to elbows; lower limbs up to knees); light touch, pain, and proprioception remain Norman F2: touch and pain sensations were absent in the lower limbs up to the knee, but normal in the upper limbs; proprioception in the upper limbs was normal, and vibration sense was decreased from distal to proximal segments
Autonomic disturbances	P1: Hyperhidrosis, urinary incontinence, P2: + P3: hyperhidrosis, slow pupillary reaction to light, Urinary incontinence	NR	Excessive sweating, orthostatic dizziness, Urge incontinence	F1: +, F2: −	F1: Recurrent vasovagal presyncope F2: −	Urinary incontinence and hyperhidrosis	F1: Both urinary and fecal incontinence F 2: urinary incontinence
Motor involvement	P1: − P2: − P3: Tetrapyramidal syndrome, Babinski signs, spastic gait and distal weakness in lower limbs	—	Proximal distal weakness in lower extremities, spastic gait	F1: Mild weakness in feet and legs, clonus in lower limbs F2: Mild spasticity in the lower limbs, pyramidal weakness	F1: Brisk DTR, Babinski sign F2: Mild lower limb weakness and absent Achilles DTR	Mute plantar response, hypoactive styloradial reflexes, absent patellar reflexes	F1: Progressive lower limb spasticity with hyperreflexia, toe‐walking, mild knee contractures F2: normal upper limb strength with a slight reduction (graded 4/5) in the lower limbs, deep tendon reflexes graded as 2+ in the upper limbs with brisk responses in the lower limbs, and a negative Hoffman sign
Variant	P1: c.926C>A;p.Ser309Ter P2: c.433C>T;p.Gln145Ter P3: c.17_18delCT;p.Pro7GlyfsTer133	c.873+2T>C	c.826delA;p.Ser276Valfs*8	F1 and F2: c.926C>A;p.Ser309Ter	F1: c.896_897delAA;p.Lys299Argfs*6 F2: c.1426del;p.Gln476Argfs*57	c.321G>A;p.Trp107Ter	F1: c.458+2T>C F2: c.458+2T>C

Motor involvement across the reviewed cases (Table 2) shows a marked clinical heterogeneity ranging from absent motor findings to prominent pyramidal signs and spastic gait. In the original series by Kurth et al. [15], motor involvement varied from absent findings in P1 and P2 to a clear upper motor neuron phenotype in P3 (tetrapyramidal syndrome with Babinski signs and spastic gait) despite an HSAN2B diagnosis, supporting that pyramidal tract features can occur in a subset of RETREG1/FAM134B‐related cases. Similarly, Aydinlar et al. [21] reported proximal/distal lower‐extremity weakness accompanied by spastic gait, and Wakil et al. [6] described intrafamilial variability with one individual showing mild distal weakness and clonus while another had mild lower‐limb spasticity and pyramidal weakness. Falcão de Campos et al. [22] also documented motor/pyramidal signs (e.g., brisk reflexes and Babinski sign) and, in another affected individual, mild lower‐limb weakness with absent Achilles reflexes, indicating that motor findings may be subtle and coexist with areflexia depending on the level of peripheral neuropathy. In contrast, the Turkish cases reported by Tasdelen et al. [5] primarily showed reflex abnormalities without a clearly described spastic phenotype (mute plantar response, hypoactive/absent reflexes), underscoring that some RETREG1‐associated presentations remain predominantly peripheral rather than pyramidal. Against this background, our index patient (F1) falls within the “spasticity‐predominant” end of the spectrum, characterized by progressive lower‐limb spasticity, hyperreflexia, toe‐walking, and contractures, whereas our affected relative (F2) demonstrates mild distal weakness with brisk lower‐limb reflexes but without definitively documented spasticity, suggesting phenotypic variability even within the same extended family. This suggests that in some HSAN IIB cases, motor dysfunction particularly spasticity may represent an early or even initial clinical clue, especially before overt neuropathic findings appear.

In contrast, the majority of other reported cases primarily featured sensory and autonomic disturbances, such as ulcerations, loss of pain and temperature sensation, and hyperhidrosis, with minimal or no motor involvement [23, 24, 25]. These differences raise the possibility of distinct phenotypic clusters within HSAN IIB, potentially influenced by the underlying mutation type or modifying genetic and environmental factors. For instance, while most variants in the reviewed cases involve nonsense or frameshift mutations in *FAM134B*, those with prominent spasticity may share certain mechanistic pathways affecting central motor tracts.

Therefore, distinguishing features between the “spastic‐predominant” and “sensory‐autonomic–predominant” subtypes could have important diagnostic implications, particularly when patients present with atypical features. The presence of early spasticity should prompt consideration of HSAN IIB in the differential diagnosis, even in the absence of early ulcers or overt neuropathy, and may justify genetic testing especially in the context of consanguinity or suggestive family history, as seen in our case (Table 2).

Three tests are crucial for diagnosing HSAN IIb: electrophysiological, histopathological, and genetic assessments. Electrophysiological testing typically shows diminished or absent sensory nerve action potentials, either preserved or reduced nerve conduction velocities. However, it is important to note that in some cases, especially in early stages, the electrophysiological findings may initially appear within normal limits. This is particularly relevant when spastic gait is an early clinical sign, as neuropathic changes on nerve conduction studies may manifest later. In our patient, the initial nerve conduction study was within the normal range, but pathological findings became evident only after a period of clinical progression.

There is no cure for HSAN, and treatment primarily focuses on preventing physical injuries and managing complications that may arise from sensory deficits. Early diagnosis, combined with careful injury prevention strategies and regular medical monitoring, can significantly improve long‐term outcomes and quality of life. Tailored footwear and thorough foot care are essential to reduce the risk of recurrent ulcers. Although individuals with HSAN IIB generally maintain a stable course and preserve ambulation with only mild gait abnormalities, some patients may develop spasticity or increased muscle tone due to overlapping or secondary neurological involvement. Medications like baclofen, a GABA agonist used to reduce spasticity, can help improve gait and reduce discomfort. However, there is no evidence or study for using baclofen in this type of neuropathy with spasticity. In our case, baclofen was prescribed to manage lower limb spasticity and enhance functional mobility; however, despite gradual dose escalation, the patient and family reported no noticeable clinical benefit. Nevertheless, the literature underscores the potential for severe complications in HSAN when patients are not closely followed by a multidisciplinary medical team.

## 5 Conclusion

In conclusion, this case broadens the clinical spectrum of RETREG1 (FAM134B)‐related HSAN2B by showing that progressive lower‐limb spasticity and pyramidal signs can be an early and predominant manifestation, potentially preceding clear sensory deficits on initial electrophysiology. Therefore, HSAN2B should be considered in the differential diagnosis of complex spastic paraplegia–like presentations, particularly in consanguineous families or when there is a suggestive family history of painless ulcers, and genetic testing can be decisive for diagnosis and counseling.

## Author Contributions

**Farzad Ahmadabadi:** supervision, data curation, writing – review and editing, conceptualization. **Marzieh Babaee:** methodology, validation, writing – review and editing, data curation. **Narjes Jafari:** data curation, supervision, investigation. **Mohadeseh Fathi:** methodology, validation, writing – review and editing. **Diba Akbarzadeh:** writing – original draft, investigation, writing – review and editing. **Amin Jazayeri:** writing – review and editing, investigation, conceptualization, writing – original draft.

## Funding

The study was conducted as part of the academic and professional activities of the authors, without external financial support.

## Consent

Written informed consent for publication of the clinical details and any accompanying images was obtained from the patient's parent/legal guardian.

## Conflicts of Interest

The authors declare no conflicts of interest.

## Data Availability

The data that support the findings of this study are available from the corresponding author upon reasonable request.
